# Miocene honey bees from the Randeck Maar of southwestern Germany (Hymenoptera, Apidae)

**DOI:** 10.3897/zookeys.96.752

**Published:** 2011-05-10

**Authors:** Ulrich Kotthoff, Torsten Wappler, Michael S. Engel

**Affiliations:** 1 Geologisch-Paläontologisches Institut, Universität Hamburg, Bundesstraße 55, D-20146 Hamburg, Germany; 2 Steinmann-Institut für Geologie, Mineralogie und Paläontologie, Universität Bonn, Nußallee 8, D-53115 Bonn, Germany; 3 Division of Entomology (Paleoentomology), Natural History Museum, and Department of Ecology & Evolutionary Biology, 1501 Crestline Drive – Suite 140, University of Kansas, Lawrence, Kansas 66049-2811, USA

**Keywords:** Apoidea, Anthophila, Apinae, *Apis*, honey bees, taxonomy, Tertiary, morphometrics

## Abstract

The Miocene Randeck Maar (southwestern Germany) is one of the only sites with abundant material of fossil honey bees. The fauna has been the focus of much scrutiny by early authors who recognized multiple species or subspecies within the fauna. The history of work on the Randeck Maar is briefly reviewed and these fossils placed into context with other Tertiary and living species of the genus *Apis* Linnaeus (Apinae: Apini). Previously unrecorded specimens from Randeck Maar were compared with earlier series in an attempt to evaluate the observed variation. A morphometric analysis of forewing venation angles across representative Recent and Tertiary species of *Apis* as well as various non-Apini controls was undertaken to evaluate the distribution of variation in fossil honey bees. The resulting dendrogram shows considerable variation concerning the wing venation of Miocene Apini, but intergradation of other morphological characters reveals no clear pattern of separate species. This suggests that a single, highly variable species was present in Europe during the Miocene. The pattern also supports the notion that the multiple species and subspecies proposed by earlier authors for the Randeck Maar honey bee fauna are not valid, and all are accordingly recognized as *Apis armbrusteri* Zeuner.

## Introduction

Honey bees (Apini Latreille: *Apis* Linnaeus) are among the most familiar of animals, with a tight association with humans since their domestication and use worldwide in agricultural ecosystems as crop pollinators (e.g., Partap 2011) and for the honey they produce. Species of *Apis*, particularly the familiar Western Honey Bee, *Apis mellifera* L., have been transported throughout the globe and are today cosmopolitan, with intensive research programs focusing on apiculture and related topics in every corner of the world. However, like most groups of Apoidea, little attention has been paid to the historical record of honey bees, outside of their most recent history since domestication. The more ancient, fossil record of *Apis* has become the focus of more critical research efforts only within the last 10–15 years (e.g., Engel 1998, 2006; Nel et al. 1999). This is partly owing to a dearth of material but also to the slow development of paleomelittology which has expanded significantly during the last two decades (Engel 2011).

The earliest definitive members of the tribe Apini are known from the Oligocene of France and Germany. These comprise *Apis henshawi* Cockerell, and under some classificatory schemes *Apis vetusta* Engel, from Rott and Enspel, Germany (e.g., Cockerell 1907; Statz 1931, 1934; Engel 1998, 1999; Wedmann 2000), and *Apis cuenoti* Théobald from Céreste, France (Théobald 1937; Nel et al. 1999), the latter of which is sometimes considered a synonym of *Apis henshawi* (Engel 1999). Generally the forewing venation of these Oligocene honey bee populations resembles that of Recent *Apis dorsata* Fabricius, but the species are distinctly smaller, more typical of the averaged-sized *Apis mellifera* and *Apis cerana* Fabricius (e.g., Nel et al. 1999, Wedmann 2000). Apini from the Oligocene and Miocene are known from Spain and France (e.g., Arillo et al. 1996, Nel et al. 1999), Italy (Handlirsch 1907), Germany (e.g., Zeuner 1931; Pongrácz 1931; Armbruster 1938a; Prokop and Fikáček 2007), Austria (Nel et al. 1999), the Czech Republic (Říha 1973; Prokop and Nel 2003), China (Hong 1983; Zhang 1989, 1990), Japan (Engel 2005, 2006), and most surprisingly the western United States (Engel et al. 2009). For most of the Late Oligocene and Miocene forms, the specific status remains questionable (Nel et al. 1999; Engel 2006). There are no unquestionable fossils of *Apis* from the Pliocene, and only records of modern *Apis mellifera* in East African copal of Late Pleistocene or younger age (e.g., Foord 1890; Cockerell 1909; Zeuner and Manning 1976) as well as petrified combs of *Apis cerana* from the Malay Peninsula (Stauffer 1979). Among all of these records, the honey bees from Rott and the Randeck Maar in Germany are the most abundant, particularly from the latter deposit.

William Scheuthle was the first to discover honey bees at the Randeck Maar (Early Miocene, southwest Germany) in 1926, and in 1928 he and Ludwig Armbruster, a prominent apiculturist of the day, excavated more material. Finally, in 1938 the accumulated material was first formally described based on an examination of 72 specimens (Armbruster 1938a, 1938b, 1938c). Armbruster (1938a) classified the material into three species of a then new genus, dubbed *Hauffapis*, although he himself pointed to the obvious similarities of *Hauffapis* to *Apis* and especially to Recent *Apis dorsata* and the contemporaneous fossil species *Apis armbrusteri* Zeuner from the nearby Böttingen Marmor (Zeuner 1931). The generic name *Hauffapis*, unfortunately, was not validly proposed and so is not nomenclaturally available (Michener 1990, 1997; Engel 1999). Armbruster (1938a) also noted that some specimens resembled *Apis mellifera* in terms of forewing venation (*vide infra*), which further convinced him that he was dealing with multiple species and which he named *Hauffapis scheuthlei*, *Hauffapis scheeri* and *Hauffapis scharmanni* (naming them for his collecting partners, along with various infraspecific forms). Subsequently, Zeuner and Manning (1976) united all of these taxa, including that from Böttingen Marmor, into a single species and under the name *Apis armbrusteri*, considering Armbruster’s three forms to be separate subspecies. The fossil bees from the Böttingen Marmor are preserved only as hollow imprints and, while they can be attributed to *Apis*, many features remain unknown from the type series (Zeuner 1931, Zeuner and Manning 1976). In order to stabilize the application of names for these bees a petition has been submitted to the ICZN to conserve universal usage of the name *Apis armbrusteri* by designation of a neotype (Engel et al. in press).

The abundance of material from Randeck Maar represents a wonderful opportunity to evaluate more critically these fossil honey bees, since from most localities only one or a very few specimens are typically available. Unfortunately, several of the diagnostics used for the determination of extant *Apis* species or subspecies cannot be used for the differentiation of fossil Apini, even when excluding the obvious biochemical attributes. For example, *Apis cerana*, *Apis mellifera*, and their subspecies, along with *Apis koschevnikovi* Enderlein and *Apis nigrocincta* Smith, are generally recognized from differences in size, coloration of setae and integument, distribution and proportions of setal bands on the metasoma, length of the proboscis, sternal and leg podite proportions, the presence or absence of a distal abscissa to M in the hind wing (absent in *Apis mellifera*), structure of the drone legs and endophallus, and behavioral aspects such as the time of drone mating flights, structure of brood cell caps, or the position of a worker while wing-flapping in front of the hive (e.g., Ruttner 1988; Verma et al. 1994; Hadisoesilo and Otis 1996, 1998; Damus and Otis 1997; Sheppard et al. 1997; Sheppard and Meixner 2003; Radloff et al. 2010, 2011). Several of these are highly variable (e.g., size, coloration, time of drone flights), while more consistent traits such as those from the hind wing are infrequently preserved in fossil *Apis*. Moreover, behavioral aspects are rarely detectable in the fossil record unless they leave a discrete trace or physical structure suitable for fossilization [e.g., traces of leaf-cutter bees (Wappler and Engel 2003; Wedmann et al. 2009), fossilized nests (Stauffer 1979)]. To date no fossil of a drone honey bee has been recovered and, indeed, male bees of any tribe or family are exceptionally rare as fossils (e.g., Camargo et al. 2000; Engel 2001a; Hinojosa-Díaz and Engel 2007). Thus, using only the typical criteria for segregating species such as *Apis cerana*, *Apis mellifera*, or their relatives, and particularly subspecies within each of these forms, it would be nearly impossible to distinguish these taxa in the fossil record. This has greatly hampered any understanding of fossil *Apis*.

In order to circumvent these extreme limitations in studying fossil Apini, herein we follow the approach of DuPraw (1965), Ruttner (1988), Rinderer et al. (1989, 1995), Wedmann (2000), and other apiculturists to analyze forewing venation angles (hereafter “FWVA”), i.e., the angles between specific landmarks (vein and crossvein bifurcations or fusions) in the forewing remigium. Given that the forewing is often very well preserved in fossil insects it permits a more meaningful comparison between Recent and fossil Apini. The approach to measure FWVA is the least complicated method for recording numerous wing characteristics (DuPraw 1965, Ruttner 1988). We agree with Engel (2006) that the recognition of taxa in Apini based solely on morphometric measurements of the forewing venation should be regarded with caution (*vide etiam* Radloff and Hepburn 1998; Hepburn 2000; Hepburn and Radloff 2002). However, forewings are one suite of morphological features that can permit the assignment of individuals to genera or sometimes even species for numerous kinds of insects, even if all other attributes are missing. For example, automatic bee identification systems that are based on forewing analyses (e.g., Steinhage et al. 2006) have met with some success. In addition, Tofilski (2008) has shown that identification of *Apis mellifera* subspecies based on forewing morphometry is >80% successful. In contrast with some other morphological features such as setal length or lengths of extremities, FWVA are probably not associated with environmental parameters such as elevation, rainfall, temperature, and latitude, as has been demonstrated for Recent populations of *Apis cerana* (Tan et al. 2003), although note that the relative proportion of presence of some wing features do occur along weak latitudinal or longitudinal clines (e.g., the proportion of individuals with an adventitious Rs2 in the forewing: Tan et al. 2008). Naturally, any consideration of fossil wings must also take into consideration possible deformation resulting from fossilization or subsequent tectonic activity. Fortunately, deformations of wing venation are relatively easy to recognize, and the approach of Rinderer et al. (1989, 1995) and Wedmann (2000), which includes the complete wing venation, is more objective and less bias-prone than the methods employed by earlier authors who studied only a few cells (e.g., Armbruster 1938a, 1938b, 1938c). Thus, despite its obvious limitations, we believe FWVA analysis is perhaps the most reliable suite of data currently available for statistically comparing living and fossil Apini.

While we are well cognizant of the fact that dendrograms resulting from cluster (phenetic) analyses cannot be equated with phylogenies owing to the inability of such methods to distinguish plesiomorphic and apomorphic features or homologies from analogies, and that these are more useful at the level of tokogenetic relationships (e.g., Hennig 1966; Wiley 1981; Schuh 2000), such analyses are nonetheless informative heuristic methods for evaluating the general similarity of populations and lineages and may provide novel insights for fossil *Apis*. Accordingly, herein we evaluate the forewing morphometrics of the Randeck Maar honey bees, including the three subspecies of Armbruster, and provide descriptive notes and analysis of previously unstudied specimens.

## Recent honey bee species

**Figure 1.**
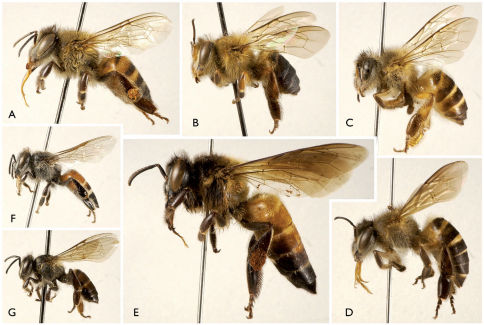
Modern honey bee diversity (all bees are workers and to the same scale). **A** *Apis mellifera* Linnaeus **B** *Apis koschevnikovi* Enderlein **C** *Apis nigrocincta* Smith **D** *Apis cerana* Fabricius **E** *Apis dorsata* Fabricius **F** *Apis florea* Fabricius **G** *Apis andreniformis* Smith. After Engel et al. (2009).

The number of Recent species of *Apis* and their respective diagnoses has been a matter of debate over the last couple of decades. Interpretations vary between six or seven species on the conservative end (Alexander 1991a, 1991b, Engel and Schultz 1997, Engel 1999: Fig. 1) and 10 or 11 (e.g., Arias and Sheppard 2005; Lo et al. 2010), or even as many as 24 (Maa 1953) at the higher extreme. Most of the controversy surrounds the status of some Southeast Asian populations (Koeniger et al. 2010; Radloff et al. 2011). While several analyses have examined *Apis* phylogeny, most recent investigations have relied solely on DNA sequence data and sometimes with exceptionally small samples across the diversity of honey bee populations (e.g., Willis et al. 1992; Tanaka et al. 2001; Arias and Sheppard 2005; Raffiudin and Crozier 2007; Lo et al. 2010). Only one analysis has synthesized data from multiple sources – adult morphology, larval morphology, DNA sequences, and behavior (Engel and Schultz 1997). The species recognized in the Engel and Schultz (1997) combined analysis were *Apis mellifera*, *Apis florea* Fabricius, *Apis andreniformis* Smith, *Apis koschevnikovi*, *Apis cerana*, and *Apis dorsata* (these authors did not consider *Apis nigrocincta* specifically distinct from *Apis cerana* at that time). *Apis nigrocincta* was subsequently added to this list of honey bee diversity (Hadisoesilo et al. 1995; Hadisoesilo and Otis 1996, 1998; Engel 1999; Smith et al. 2000, 2003) (Fig. 1). While the species recognized in the diversity of phylogenetic treatments varies under the biological, phylogenetic, or evolutionary species concepts, there remains broad congruence as to the principal clades within the genus and their interrelationships (e.g., Engel and Schultz 1997; Engel 1998, 1999, 2006; Leelamanit et al. 2004; Arias and Sheppard 2005; Raffiudin and Crozier 2007; Lo et al. 2010). These studies agree that the lineage of dwarf honey bees, *Apis florea* and *Apis andreniformis*, diverged early on from the remainder of Recent *Apis* clades, with the giant honey bees, *Apis dorsata* and its predecessors, diverging from the common ancestor of a clade comprising *Apis mellifera* and the “*cerana*” group of species (i.e., *Apis cerana*, *Apis koschevnikovi*, *Apis nigrocincta*). These three groups are sometimes accorded subgeneric status as *Micrapis* Ashmead, *Megapis* Ashmead, and *Apis* s.str. (e.g., Engel 1999, 2001b, 2002, 2006; Engel et al. 2009; Koeniger et al. 2011), although some less widely employed classifications have considered them as separate genera in their own right (e.g., Ashmead 1904; Maa 1953; Wu and Kuang 1987). *Apis mellifera* is the most widespread of these species, occurring throughout Europe, Africa, northernwestern Asia (e.g., Ponto-Caspian and as far East as the Tien Shan), the Levant, Caucasia, and the Iranian Plateau (Ruttner 1988, 1992; Ruttner et al. 1985; Sheppard and Meixner 2003), as well as adventive in the Americas and Australia (e.g., Kerr 1957; Sheppard 1989; Engel 1999; Moritz et al. 2005). The remaining Recent honey bees are largely restricted to Asia (Michener 2007; Radloff et al. 2011), with the exception of *Apis florea* which is known also from Jordan, the eastern Arabian Peninsula, and northeastern Africa (Lord and Nagi 1987; Mogga and Ruttner 1988; Engel 1999; Michener 2007; Dathe 2009; Haddad et al. 2009; Moritz et al. 2010). The precise distributions of the remaining Asian species and morphotypes are summarized by Otis (1996), Engel (1999), Oldroyd and Wongsiri (2006), and Hepburn and Radloff (2011). We did not attempt to evaluate the morphometrics of the complete suite of forewing variation in modern *Apis* species, which is well beyond the scope of the current study. Instead, for the purposes of our analyses (*vide infra*), it was most critical to simply represent the broadest sample of variation across the genus. Accordingly, we employed representatives of the three principal clades, or subgenera, of *Apis*.

## Geological setting

The Randeck Maar is located in southwest Germany, southeast of Stuttgart at the escarpment of the Swabian Alb (48°71'N, 9°31.8'E, 750m elevation) and originated during the Miocene. During this epoch, the Mesozoic rocks of the Swabian Alb were penetrated by numerous volcanic dykes leading to phreatomagmatic eruptions when the rising nepheline-melilithitic magma contacted groundwater (Bleich 1988). The Maar deposits consist of volcanoclastic limestones overlain by Miocene sediments (Krautter and Schweigert 1991), which are dated as Early/Middle Miocene (Burdigalian, Karpatian, MN 5, ca. 16–18 Ma) after the mammal fauna (Heizmann 1983). In one phase of sedimentation, bituminous laminites (‘dysodiles’) and laminated, varve-like limestones were deposited. These limestones contain exceptionally well preserved fossil insects (e.g., Armbruster et al. 1938a, 1939; Schawaller 1986; Kotthoff 2005; Kotthoff and Schmid 2005).

## Material and methods

**Figure 2.**
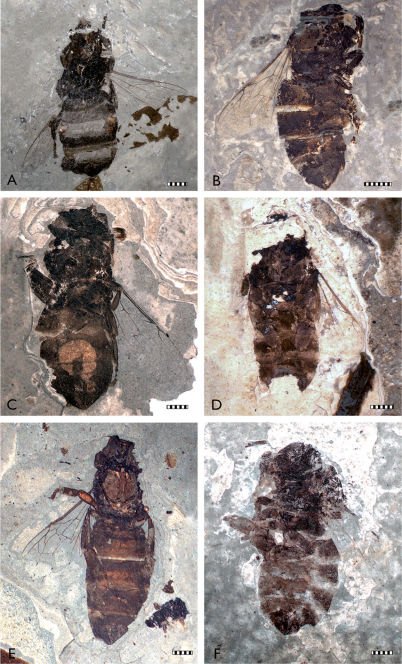
Photomicrographs of representative Randeck Maar honey bees (*Apis armbrusteri* Zeuner). **A** SMNS 64675 (neotype) [Morphotype D] **B** SMNS 64674/12 [Morphotype D] **C** SMNS 64674/11b [Morphotype D?] **D** SMNS 64674/11a [Morphotype CM] **E** SMNS 64674/21 **F** SMNS 64674/28. Scale bar = 2 mm.

**Figure 3.**
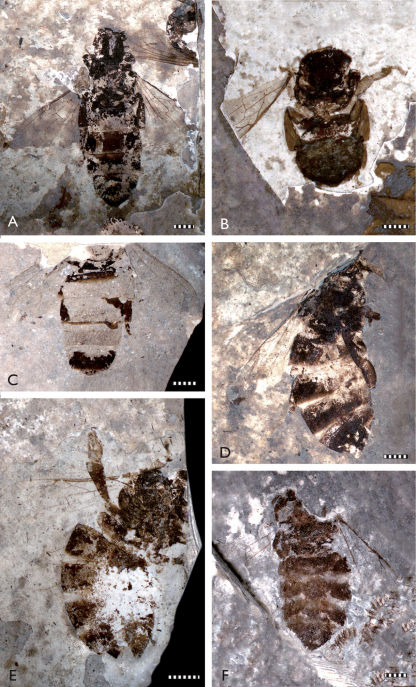
Photomicrographs of representative Randeck Maar honey bees (*Apis armbrusteri* Zeuner). **A** SMNS 64674/18 [Morphotype D] **B** SMNS 64674/49 [Morphotype D] **C** SMNS 64674/30 [Morphotype D] **D** SMNS 64674/35 [Morphotype D] **E** SMNS 64674/19 [Morphotype D] **F** SMNS 64674/36 [Morphotype CM]. Scale bar = 2 mm.

**Figure 4.**
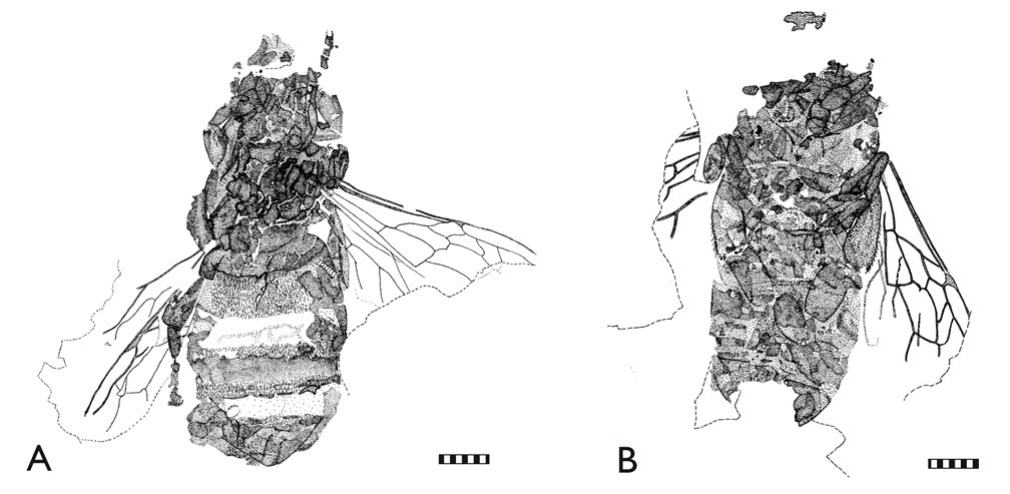
Representative Randeck Maar honey bees (*Apis armbrusteri* Zeuner). **A** SMNS 64675 (neotype) [Morphotype D] **B** SMNS 64674/11a [Morphotype CM]. Scale bar = 2 mm.

**Figure 5.**
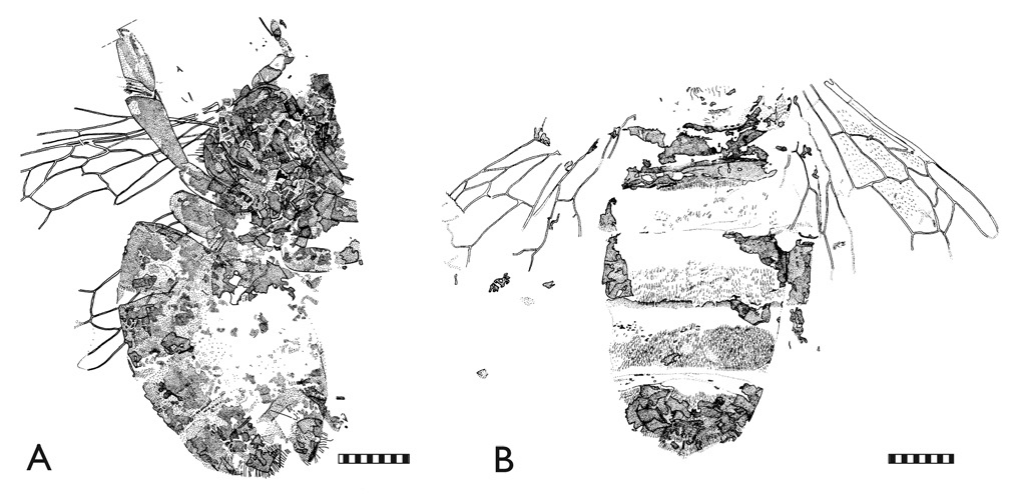
Representative Randeck Maar honey bees (*Apis armbrusteri* Zeuner). **A** SMNS 64674/19 [Morphotype D] **B** SMNS 64674/30 [Morphotype D]. Scale bar = 2 mm.

The fossil material studied originates from the Staatliches Museum für Naturkunde, Stuttgart (SMNS), the Heimatmuseum Göppingen Jebenhausen (HMJ), and the Paläontologisches Museum Nierstein (PMN) (Figs 2–5). Additional *Apis armbrusteri* specimens are present in the Urwelt-Museum Hauff but were already considered in detail by Armbruster (1938a). A re-examination of the majority of the specimens described in Armbruster (1938a) was impossible since many of these were covered in Canada balsam, ironically used by Armbruster to preserve the bees (Armbruster 1938a, 1939), but which has darkened over time. Removing the balsam likely will lead to the destruction of many important features. In total, 18 not yet described specimens of *Apis armbrusteri* are introduced in this work (Table 1).

Fossils were examined with a stereomicroscope, while drawings were prepared using a camera lucida. Photographs were taken with a digital camera and the software “Analysis Pro Version 3.1” (SIS) used for distance and angle measurements. The software “PAST” version 1.75b (Hammer et al. 2001) was used for cluster analyses. Nomenclature of wing veins and cells follows that of Engel (2001a), while landmarks and angles for FWVA analysis follow those of previous authors (e.g., Ruttner 1988; Rinderer et al. 1989, 1995; Wedmann 2000).

FWVA measurements of all specimens documented herein and with well-preserved forewings were subjected to a cluster analysis together with measurements of representative Recent Apini from Europe (*Apis mellifera*; eleven specimens) and Asia (*Apis florea* Fabricius; four specimens; *Apis dorsata*; twelve specimens; *Apis cerana*; 14 specimens). So as to expand our comparative treatment we included other Miocene and Oligocene honey bees that had sufficiently well-preserved forewings to permit meaningful measurement and comparison. These included the European material of *Apis henshawi* (ten specimens; Cockerell 1907; Wedmann 2000); *Apis cuenoti* (two specimens; Théobald 1937; Nel et al. 1999); Arillo et al. (1996) and Nel et al. (1999) Oligocene and Miocene *Apis aquisextana* (two specimens; erroneously as “*Apis aquisextusensis*” in the latter publication: Engel 2006), and forms B, C, E, F, G, H, I, and J (twenty specimens; Nel et al. 1999); *Apis lithohermaea* Engel from Japan (Engel 2006); and *Apis nearctica* Engel et al. from North America (Engel et al. 2009). For comparative purposes, we included FWVA measurements from other Eocene Apidae (*Electrapis* Cockerell, *Electrobombus* Engel, *Succinapis* Engel, *Thaumastobombus* Engel, *Melikertes* Engel, and *Pygomelissa* Engel and Wappler; one or two specimens per taxon), as well as other tribes of Recent corbiculate (*Bombus* Latreille, *Euglossa* Latreille, *Eufriesea* Cockerell; one specimen each) and non-corbiculate Apinae (*Centris* Fabricius, *Epicharis* Klug, *Xeromelecta* Linsley, and *Zacosmia* Ashmead; one specimen each). In total, 97 forewings were analyzed, and additionally, 19 measurements taken by Wedmann (2000) were added for the cluster analysis (see Appendix I).

While it would have been ideal to make the analysis more robust with the inclusion of more of Armbruster’s original material, this was not possible. Most of the specimens described by Armbruster (1938a) are now lost and many of the few remaining are rendered useless for examination owing to the unfortunate application of Canadian balsam (Armbruster 1939). As such, only Armbruster’s (1938a) photographs and illustrations were of use.

Although the venation of drones does not differ significantly from that of workers, in order to completely eliminate potential caste differences two drones of *Apis cerana* from Pakistan were added to the analysis as a control. While the drones were separated from workers, they were still more similar to conspecific workers than to specimens of any other taxon. Further tests which included drones of *Apis mellifera* found similar results (Kotthoff 2002). Thus, gender did not introduce any bias into the results even though all fossil *Apis* discovered to date are workers (Engel 1998, 2006; Nel et al. 1999; Engel et al. 2009; [Bibr B31]herein).

**Table 1.** List of not-yet described specimens of *Apis armbrusteri* presented in this work.

*Museum/number*		*body length (mm)*	*forewing length (mm)*	*hindwing length (mm)*	*Sediment/Annotations*
Wing venation not well preserved:					
HMJ	A 817	12.6	7.5	-	light varve layer
SMNS	64674/17a	21.6	-	-	dark grey limestone
SMNS	64674/21a/b	16.9	8.9*	6.0*	dark grey limestone
SMNS	64674/28	15.0	-	-	dark varve layer
SMNS	64674/31	13.9	-	-	dark varve layer
SMNS	64674/38	16.0	-	-	light limestone
SMNS	64674/50a	24.3	>15	-	dark varve layer
PMN	SSN10RM12	16.9	9.1	-	light varve layer
Wing venation well preserved:					
SMNS	64674/11a	13.2	>7.9	-	light varve layer
SMNS	64674/11b & 11c	14.1	>8.7*	-	dark varve layer
SMNS	64674/12a & 12b	15.7	9.9	7.3	dark grey calcareous marl
SMNS	64674/18	17.4	9.7*	7.8*	dark varve layer
SMNS	64674/19	17.0	>10.3	-	dark varve layer
SMNS	64674/30	-	8.4	-	dark varve layer
SMNS	64674/35	>15.0**	>11.2		dark varve layer
SMNS	64674/36	>14.7**	10.0		dark varve layer
SMNS	64674/49	9.9**	>9.0		light varve layer
SMNS	64675	14.3	8.1		dark varve layer

* distal part reconstructed based on similar complete wings of other specimens

** head missing

## Systematic paleontology

### Genus Apis Linnaeus Apis armbrusteri Zeuner

Refer to Engel et al. (2009) for a complete taxonomic summary for the species, and to Engel et al. (in press) for details on the neotype (SMNS 64675: Figs 2A, 4B). Herein we provide descriptive notes for a series of specimens not previously documented by earlier authors. For those that are most completely preserved we have noted whether the specimens are of a *cerana*/*mellifera*-like morphotype (CM) or a *dorsata*-like morphotype (D). All metrics are provided in millimetres.

### Specimens with poorly-preserved or missing wings

**HMJ A 817.** *Metrics*: body length 12.6; metasoma 7.3; mesosoma 3.2; head 1.8; forewing 7.5. *Descriptive notes*: Ventral view on light varve layer; preservation exceptionally poor; legs missing except for a few fragments, apparently preserving only metatibiae, which are relatively long and slender; wings only fragmentarily preserved, fragments match forewings of other *Apis armbrusteri*; no counterpart.

**SMNS 64674/17a**. *Metrics*: body length 21.6. *Descriptive notes*: Ventral view on dark grey limestone; fossil re-crystallized and fragmented; metasoma highly deformed and obviously swollen, resulting in extra-ordinary high body length; forewing venation poorly preserved; left hind leg positioned parallel to metasoma, revealing a slender metabasitarsus; head ventrally directed; compound eyes small in proportion to head and far apart from each other; counterpart is SMNS/17b, which is even more poorly preserved.

**SMNS 64674/21a.** *Metrics*: body length 16.9; metasoma 9.9; mesosoma 4.7; head 2.8; mesofemur 1.2; mesotibia 1.6; mesobasitarsus 1.6; metafemur 2.1; metatibia 3.2; metabasitarsus 2.7; forewing (reconstructed) 8.9; hind wing (reconstructed) 6.0.*Descriptive notes*: Ventral view on dark grey limestone; parts of dorsal cuticle apparent; right forewing well preserved, but folded; metasoma very long (probably resulting from swelling in water after death) and well preserved, but not completely exposed from matrix; sting apparatus apparent; left metatibia long and slender; metabasitarsus very long and broadened; setae preserved in some areas of metabasitarsus; glossa and galeae evident between mandibles; counterpart is SMNS 64674/21b.

**SMNS 64674/28.** *Metrics*: body length 15.0; metasoma 9.0; mesosoma 4.7; head 2.1; glossa 2.9; profemur 2.2; protibia 1.6; probasitarsus 1.3; mesofemur 2.3; mesotibia 1.9; metabasitarsus 2.5. *Descriptive notes*: Lateral view of inner surface on dark varve layer; fossil slightly turned ventral; right forewing venation only partly visible; fragment of left forewing preserved; metatibia and metabasitarsus appear flattened and short; mesoscutum broken; mandibles well preserved; glossa appears protruded; counterpart (SMNS, not registered) exhibits a few dorsal elements, fragments of left forewing, and parts of the other lateral side.

**SMNS 64674/31.** *Metrics*: body length 13.9; metasoma 7.6; mesosoma 3.8; head 3.1; metafemur 2.8; metatibia 2.9; metabasitarsus 1.8. *Descriptive notes*: Laterally embedded on dark varve layer; head turned upwards; mandibles well preserved; right hind leg exposed above metasoma; metatibia and metabasitarsus appear short and broadened; metabasitarsus partly covered by metasoma; wings not preserved; no counterpart.

**SMNS 64674/38.** *Metrics*: body length 16.0; metasoma 9.0; mesosoma 5.5; head 2.8; metatibia 2.7; metabasitarsus 1.8. *Descriptive notes*: Laterally embedded on light limestone; fragmentary preservation of head and mesosoma; metabasitarsus of presumably left hind leg flat and short; metasoma obviously swollen and compressed; wings not preserved.

**SMNS 64674/50a.** *Metrics*: body length 24.3; forewing >15; mesosoma+head 8.8. *Descriptive notes*: Largest specimen known among Miocene honey bees from Randeck Maar; preserved on dark varve layer; metasoma, especially first metasomal segment, very well preserved; mesosoma poorly preserved except for slightly arched mesoscutum; legs missing; wings oriented parallel to metasoma, wing venation not apparent; compound eyes especially well preserved; counterpart is SMNS 64674/50b. *Remarks*: Even though the metasoma may have swollen due to postmortem processes, the specimen is extraordinarily large. Additionally, the forewing length indicates that this specimen approximated an *Apis dorsata* worker in size. The average forewing length of the specimens presented here is 9.6 mm, thus the wing of SMNS 64674/50 is >50% longer. The second largest forewing length of specimen SMNS 64674/35 is more than 3 mm shorter. Due to this difference in size, we believe SMNS 64674/50 may have been an *Apis armbrusteri* queen. According to Ruttner (1988), in Recent Apini the size difference between the female castes are greatest in the dwarf honey bee *Apis florea* (worker: 6.26±0.10 mm forewing length, queen: ~35% longer), slightly less in *Apis cerana* (worker: 7.54±0.14 mm forewing length, queen: ~24% longer) and significantly lower in *Apis mellifera* and the giant honey bee *Apis dorsata*. An unequivocal identification of SMNS 64674/50 as a queen is not possible. However, we believe that the occurrence of a queen among the >90 *Apis* specimens from the Miocene Randeck Maar is more probable than the presence of one isolated giant honey bee worker. SMNS 64674/50 may be the first honey bee queen from the fossil record. The specimen is not a drone as evidenced by the relatively small size of the compound eyes rather than the nearly holoptic eyes of male honey bees.

**PMN SSN10RM12.** *Metrics*: body length 16.9; metasoma 10.0; mesosoma 4.8; head 2.5; mesofemur 1.5; mesotibia 1.8; metafemur 2.2; metatibia 2.7; metabasitarsus 1.9; scape (reconstructed) 0.6; flagellum 2.6; forewing 9.1. *Descriptive notes*: Dorsoventrally embedded and very well preserved on light varve layer; antennae very well preserved; third submarginal cell of forewing broader than that of *Apis cerana* or *Apis mellifera* (unfortunately the wings could not be analyzed in detail); metatibiae and metabasitarsi rather slender; specimen somewhat resembles *Apis henshawi*.

### Specimens with well preserved forewings

**SMNS 64674/11a [Morphotype CM].** *Metrics*: body length (reconstructed) 13.2; metasoma 7.0; mesosoma 3.7; head 2.4; mesofemur 2.1; metafemur 1.7; metabasitarsus 2.3; forewing >7.9 (most distal part not apparent). *Descriptive notes*: Dorsoventrally compressed, ventral view on light varve layer (Figs 2D, 4B); posterior metasoma not preserved, head only partially preserved; a fragment, possibly part of head, lies next to specimen; mesosoma, left forewing, and legs completely preserved; extremities ventrally exposed; one setal row of left metabasitarsus evident, number of setae estimated at 28–30; metatibia and metabasitarsus laterally flattened and short in proportion to width; some setae of rastellum preserved at metatibial apex; forewing with third submarginal cell extraordinarily long; 1m-cu with a small distal process in second medial cell; hind wing with a distal process of vein M preserved; no counterpart. *Remarks*: This specimen is small in comparison to other bees from the Randeck Maar. The forewing length of SMNS 64674/11a (>7.9, but probably not >8.5 mm) is similar to a small *Apis cerana* worker (7.5–9 mm) and shorter than the average forewings from the Miocene honey bees from Randeck (9.6 mm). The long third submarginal cell is reminiscent of *Apis cerana. A. mellifera*, and their relatives, but the process of 1m-cu is not present in these modern species, while this aberrant veinal stub appears in several individuals of *Apis armbrusteri*. The distal process of M in the hind wing does not occur in *Apis mellifera* but is present in all other Recent *Apis* species (Alexander 1991a; Engel 1999). The number of setae (28–30) in one of the setal comb rows of the metabasitarsus is similar to *Apis mellifera* (Maa 1953). *Apis mellifera* has ten setal comb rows, of which the median has about 30 setae (Maa 1953; Nitschmann and Hüsing 1987; Lutz 1993). By contrast *Apis henshawi* only has 24 setae in the medial comb (Zeuner and Manning 1976). In this regard, the specimen is more similar to *Apis mellifera* and the ‘*cerana*’ group than to *Apis dorsata* and *Apis henshawi*. Accordingly we ascribe the specimen to the CM morphotype.

**SMNS 64674/11b [Morphotype D?].** *Metrics*: body length 14.1; metasoma 8.5; mesosoma 3.5; head 2.5; metatibia 2.2; metabasitarsus 1.9; forewing (reconstructed) > 8.7; mandible 1.5. *Descriptive notes*: Dorsoventrally compressed on dark varve layer (Fig. 2C); anterior part (head in particular) slightly rotated around axis of body length; mandibles, compound eyes, and antennae well preserved; antennae with at least nine, perhaps 10, flagellomeres preserved; parts of the legs compressed close to body; left hind leg positioned lateral of metasoma; metatibia and metabasitarsus do not appear flattened or shortened in respect to homologous appendages of SMNS 64674/11a; part of metasoma re-crystallized, probably pyritized; parts of sting apparatus apparent; forewing 1m-cu with a very short process; counterpart is SMNS 64674/11c. *Remarks*: The wing venation of this specimen is generally similar to that of *Apis dorsata*; however, the third submarginal cell is not completely preserved. The shape and relative length of the metatibia and metabasitarsus are also similar to those of *Apis dorsata* but the specimen is smaller than typical workers of this species.

**SMNS 64674/12b [Morphotype D].** *Metrics*: body length 15.7; metasoma 9.7; mesosoma 4.3; head 1.8; profemur 1.5; protibia 1.5; probasitarsus 1.1; mesofemur 1.6; mesotibia 1.6; metatibia 2.4; metabasitarsus 1.8; forewing 9.9; hind wing 7.3. *Descriptive notes*: Laterally compressed on dark grey calcareous marl (Fig. 2B); body parts well preserved except for head; wing venation outstandingly well preserved; third submarginal cell rather short and meeting 2m-cu strongly distad; 1m-cu broken, with a short medioapical process projecting into second medial cell; mesosomal cuticle partly fragmented; distal five segments of metasoma stressed horizontally owing to postmortem processes; metabasitarsi fragmented but revealing a relatively slender shape; setae clearly preserved distally on metasoma; counterpart 64674/12a not preserving further details. *Remarks*: The shape of the short submarginal cell and relatively slender shape of the metabasitarsi are reminiscent of those of *Apis dorsata*. While the length of the forewing exceeds the typical length of *Apis mellifera*, it does not reach the length of *Apis dorsata*.

**SMNS 64674/18 [Morphotype D].** *Metrics*: body length 17.4; metasoma 10.2; mesosoma 3.8; head 3.3; metafemur 2.4; metatibia 3.2; metabasitarsus 2.3; metabasitarsal width 0.9; forewing (reconstructed) 9.7 (minimal); hind wing (reconstructed) 7.8 (minimal). *Descriptive notes*: Ventral aspect on dark varve layer, very well preserved but metasoma probably swollen during rest in water column and subsequently compressed during compaction, with metasoma appearing artificially lengthened and broadened; specimen length was probably ~15 mm in life (reaching general size of *Apis dorsata*); forewing venation well preserved (Fig. 3А); third submarginal cell short and broad; process of 1m-cu present; rastellum of left metatibia apparent; setal comb rows of right metabasitarsus consist of 20–25 setae; left metabasitarsus shorter and broader than that of SMNS 64674/11b but more slender than that of 64674/11a; sting very well preserved; no counterpart; next to head is wing of *Bombylius* sp. (Kotthoff 2005). *Remarks*: The short, broad third submarginal cell is similar to that of *Apis dorsata* and *Apis henshawi*. The number of setae in the setal comb rows of the right metabasitarsus are also somewhat similar to that of *Apis henshawi* (Lutz 1993).

**SMNS 64674/19 [Morphotype D].** *Metrics*: body (without head) 17.0; metasoma 8.3; mesosoma 5.6; metatibia 2.9; metabasitarsus 2.3; forewing (reconstructed) >10.3. *Descriptive notes*: Partly laterally, partly dorsoventrally compressed on dark varve layer (Figs 3Е, 5А); head missing; right hind leg extended laterally; right metatarsus not completely excavated from matrix; rastellum well preserved; wing venation of right forewing well preserved except for apicalmost area; third submarginal cell short; 1m-cu with long medioapical process projecting into second medial cell; one hind wing compressed under right forewing; distal abscissa of M apparently present; left forewing obscured by metasoma; sting preserved but sting device not apparent owing to re-crystallization in center of metasoma; no counterpart. *Remarks*: The body size of this individual is quite large and cannot be explained solely by broadening and lengthening of the metasoma from postmortem swelling given that the terga are positioned close to and largely overlapping each other. The right forewing has a short, broad third submarginal cell somewhat similar to *Apis henshawi*, with a general length presumably reaching a similar proportion to that of *Apis dorsata*. The forewing is generally similar to the forewing of SMNS 64674/18. The metabasitarsus is relatively slender.

**SMNS 64674/30 [Morphotype D].** *Metrics*: metasoma 9.1; metabasitarsus 1.8; forewing 8.4. *Descriptive notes*: Parts of metasoma and fragments of mesosoma, legs and wings preserved on dark varve layer (Figs 3Ц, 5B); metasoma mainly represented by setae and tergal fragments, presumably ventral view of dorsal elements; no counterpart. *Remarks*: The wings are similar to those of *Apis dorsata* and *Apis henshawi*, and the fragments of the hind legs indicate that the metatibia and metabasitarsus were slender, similar to those of *Apis dorsata*.

**SMNS 64674/35 [Morphotype D].** *Metrics*: body length (metasoma + mesosoma) 15.0; metasoma 9.5; mesosoma 4.8; metatibia 3.3; metabasitarsus 1.9; forewing >11.2. *Descriptive notes*: Laterally compressed, with head missing, on dark varve layer (Fig. 3D); mesosoma fragmentarily preserved; mesoscutum turned upwards; one forewing well preserved in basal part; one hind leg obscured by dorsal part of metasoma; other hind leg positioned on top of ventral part of metasoma; metatibia slender; metabasitarsus apparently broad and short, but more slender than that of SMNS 64674/11a. *Remarks*: The complete forewing was perhaps 12 mm long in life and therefore as long as an *Apis dorsata* forewing.

**SMNS 64674/36 [Morphotype CM?].** *Metrics*: body (metasoma + mesosoma) 14.7; metasoma 9.7; mesosoma 4.9; forewing (reconstructed) 10.0. *Descriptive notes*: Laterally compressed, with head missing, on dark varve layer; mesosoma and hind legs only fragmentarily preserved; right (presumably, could be left) metabasitarsus positioned along ventral part of metasoma, very broad and short; metasoma not well preserved; terga not in contact with each other; one forewing very well preserved (Fig. 3F), revealing long third submarginal cell; distal absicssa of vein M apparently present in hind wing; no counterpart. *Remarks*: The forewing of this specimen is very similar to those of *Apis cerana* and *Apis mellifera*. The shape of the metabasitarsus is also reminiscent of these species but possibly the metabasitarsus was deformed during fossilization. The size of the specimen exceeds the typical size of both *Apis cerana* and *Apis mellifera*.

**SMNS 64674/49 [Morphotype D].** *Metrics*: body length (without head) 9.9; metasoma 4.7; mesosoma 4.6; mesofemur 1.9; mesotibia 1.6; mesobasitarsus 1.6; metafemur 1.8; metatibia 2.9; metabasitarsus 1.6; forewing (reconstructed) >9.0. *Descriptive notes*: Interior apparent in ventral view on light varve layer (Fig. 3B); head not preserved; terga still connected (indicating that metasoma was barely swollen); terga slightly laterally inflected; sting apparatus well preserved; setae of a single setal comb row evident in basal part of left mesobasitarsus, consisting of ~25 setae; metabasitarsus slender and somewhat triangular in shape; forewing venation preserved except for distalmost part; no counterpart. *Remarks*: While the lengths of the mesosoma and forewings are comparable to those of other bees from the Randeck Maar, the metasoma is noticeably shorter. The forewing length and venation appear similar to that of *Apis dorsata*. In addition, the slender metabasitarsi are reminiscent of *Apis dorsata*, even though the specimen does not approximate this species in size.

**SMNS 64675 (neotype) [Morphotype D].** *Metrics*: body length 14.3; metasoma 8.1; mesosoma 3.4; head 2.6; metatibia 2.4; metabasitarsus 1.1; forewing 8.1. *Descriptive notes*: Dorsoventrally compressed on dark varve layer (Figs 2A, 4A); sterna fragmentarily preserved, setae of sterna nearly completely preserved; wax mirrors apparent as orange-brown areas (cf. Ansorge and Kohring 1995: fig. 5.1), evident on third metasomal sternum; mesosoma revealing dorsal elements; legs fragmentarily preserved; hind legs positioned next to metasoma; very slender metatibiae and metabasitarsi; metabasitarsi probably not completely preserved; some wing areas not apparent, but all cells of right forewing visible; compound eyes evident; clypeus and frons not discernible (gap in matrix separates parts of mandibles and right protarsus from remainder of specimen); fossil leaf preserved behind metasoma; another leaf positioned in same varve layer at right side of bee; wing not preserved in contact zone of leaf and wing, perhaps result of earlier preparation. *Remarks*: The forewing of this individual was probably longer than 8.1 mm, presumably reaching a length of 9.5–10 mm, and is similar to that of *Apis dorsata*. Although all phylogenetic evidence indicates that fossil *Apis* built combs like their modern counterparts (Ruttner 1988; Engel 1998), the wax mirrors confirm that the honey bees from the Miocene Randeck Maar constructed combs. The presence of leaves in the same layer may indicate that the specimen died in Autumn.

## Results

**Figure 6.**
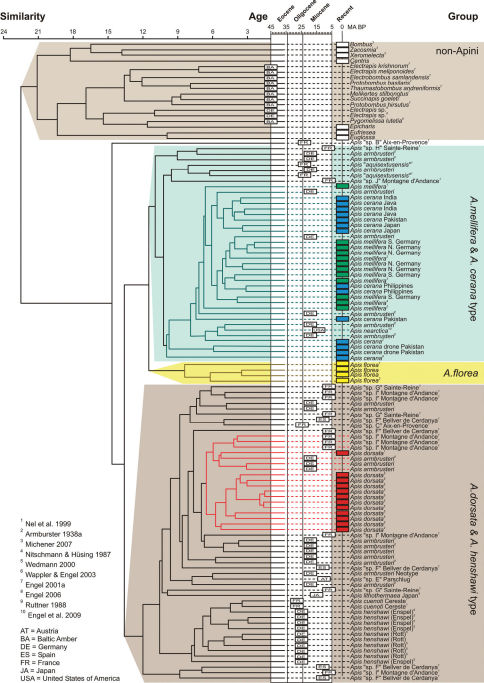
Dendrogram resulting from FWVA cluster analysis described in the text. Recent specimens of *Apis* and associated clusters are marked in the following colors: red, *Apis dorsata* Fabricius; yellow, *Apis florea* Fabricius; green, *Apis mellifera* Linnaeus; blue, *Apis cerana* Fabricius; cyan, *cerana*/*mellifera*, morphotype.

As to be expected given the unique venation of honey bees, all non-Apini were grouped together relative to *Apis* in the FWVA cluster analysis (Fig. 6). Among the Apini, all Recent forms were grouped in general accordance with their systematic position, and independent of whether the measurements were based on the literature or newly measured forewings. This underlines both the utility of the method and the quality of metrics and drawings made by different authors (e.g., Armbruster 1938a; Nel et al. 1999; Engel 2006).

The dendrogram (Fig. 6) reveals two main clusters, the first of which comprises the FWVAs of *mellifera*/*cerana* and, on a subbranch of their own, the specimens of *Apis florea*. The second major cluster consists of FWVAs of *Apis dorsata* and *Apis henshawi*, with both species well segregated from each other. These groups do not necessarily represent clades given that undoubtedly some grouping is based on symplesiomorphies. In regard to the specimens of *Apis armbrusteri*, most specimens group with *Apis dorsata*, while some specimens (e.g., SMNS 64674/11a and SMNS 64674/36) are positioned within the *cerana*/*mellifera* group (Fig. 6). The dendrogram supports the observations described above, that at least some of the specimens newly documented herein are superficially more similar in forewing venation to *cerana*/*mellifera*-like beesthan to *Apis dorsata*, the latter phylogenetically outside of the *Apis* s.str. clade (Engel and Schultz 1997; Engel 2006; Raffiudin and Crozier 2007; Lo et al. 2010).

*Apis henshawi* from the Oligocene grouped nearest to those *Apis dorsata* and *dorsata*-like fossils (Fig. 6). Not surprisingly, *Apis cuenoti* from the Oligocene of Céreste groups within *Apis henshawi*, generally supporting the synonymy of these taxa (Engel 1999).

The probably Late Oligocene-aged specimens from Aix-en-Provence, however, showed a different clustering pattern. Some of the specimens of the debated species “*Apis aquisextana*” (Arillo et al. 1996; Nel et al. 1999; Engel 2006) grouped with the *mellifera*/*cerana* branch, while another specimen (“B” of Nel et al. 1999) grouped outside all other Apini. However, Nel et al. (1999) noted that for this material the apical portions of the wings were destroyed and it is, therefore, very possible that the wing venation was altered by postmortem processes. As such, this specimen may represent merely a damaged individual of *Apis armbrusteri* of the “CM” morphotype and its current clustering position should be considered dubious. A fourth specimen (“C” of Nel et al. 1999) was positioned on a branch together with other Miocene bees whose FWVA are generally similar to *Apis dorsata* and *Apis armbrusteri* of the “D” morphotype (Fig. 6).

The Apini from the Miocene are, independent of their geographical origin, scattered across the principle clusters (i.e., the branches on which either *mellifera*/*cerana* or *Apis dorsata* and *Apis henshawi* are positioned). In addition to the specimens SMNS 64674/11a and 64674/39, six other individuals from the Miocene of Randeck showed a forewing venation more similar to that of *cerana*/*mellifera*-like bees than to that of *Apis dorsata*. The distribution of Armbruster’s specimens (based on his 1938a photographs and figures) in the dendrogram was independent of the “*Hauffapis*”-species designated by Armbruster (1938a). For example, forewings of “*Hauffapis scheuthlei*” occur next to both the *cerana/mellifera* and *Apis dorsata* clusters, indicating that the subdivisions of *Apis armbrusteri* into the several species or subspecies as advocated by Armbruster (1938a) and Zeuner and Manning (1976) are effectively meaningless. The pattern was the same for the other European Miocene fossillagerstätten, namely that the *Apis* from Montagne d’Andance and Sainte-Reine (Miocene, France) are positioned on both major branches as for the material from Randeck (Fig. 6).

## Discussion

Naturally, as noted previously, dendrograms cannot be interpreted as phylogenies owing to an inability to distinguish homology from analogy and plesiomorphy from apomorphy. This is immediately evident in that, while all *Apis* group together, the corbiculate Apinae do not form a cluster, nor do the Centridini, or other well-defined taxa based on larger suites of characters (Fig. 6). Moreover, *Apis florea* phylogenetically lies outside of an *Apis* s.str.+*Megapis* clade (Alexander 1991a, 1991b; Engel and Schultz 1997; Engel 2006; Raffiudin and Crozier 2007; Lo et al. 2010), while nonetheless sharing more plesiomorphic similarities in FWVA with *Apis* s.str. relative to *Megapis* and thereby resulting in the grouping of *Micrapis* with *Apis* s.str. in a cluster analysis (Fig. 6). Despite this inability to equate the dendrogram with a phylogeny, the FWVA analysis supports the general systematic division of the Oligocene and Recent honey bees as well as the three principle lineages of modern *Apis* (*Megapis. Micrapis*, and *Apis* s.str.) (e.g., Engel 1998, 1999, 2006).

The newly documented honey bees from the Randeck Maar exhibit, similar to the specimens described by Armbruster (1938a), a considerable variability in size, body shape, and forewing venation. Among the ten specimens newly considered in detail, two are remarkably similar to the *cerana/mellifera* group. Specimen 64674/11a in particular probably could not be differentiated from a fossil of *Apis cerana* in many respects, although the presence of the small process of 1m-cu is not found in the former species. However, other specimens such as 64674/19 are seemingly more similar to *Apis henshawi* and *Apis dorsata*, but putatively only in plesiomorphic features. However, there are also specimens for which the assignment to one or the other of the two morphotypes employed herein must remain questionable and, in general, when characters other than wing venation are examined there is gradation between these morphological extremes. It is thus not entirely clear whether the environment around the Randeck Maar hosted two different *Apis* species [or even more, as suggested by Armbruster (1938a)], or only one variable species, a phenomenon known in modern taxa such as *Apis mellifera*, *Apis cerana*, *Accipiter tachiro* (Daudin) (e.g., Hepburn and Radloff 1998; Radloff et al. 2010; Louette 2007), and particularly for variable wing morphologies in species of lower termites, bark lice, halictine bees, and many other insect lineages (e.g., Emerson 1933; Coaton 1949, 1958; Kučerová 1997; Grimaldi et al. 2008; Gibbs 2010). A clear division into two species would make sense if there were other supporting characters (e.g., leg shape, size, hind wing venation). Though, as noted, this cannot be demonstrated for any of the bees from the Miocene of Randeck. Additionally, other European sites also show the presence of different morphotypes within the same locality, particularly Montagne d’Andance and Sainte-Reine (Miocene, France) (*vide supra*). Conversely, Recent honey bees in Asia, while broadly overlapping, tend not to occur in the same microhabitats. For example, *Apis dorsata* is more common at higher elevations, and *Apis florea* uses a special ecological niche in the stratum of dense bushes and small trees in tropical areas (e.g., Wu and Kuang 1987; Ruttner 1988). *Apis mellifera* and *Apis cerana* do not occur in the same regions naturally, and where *Apis mellifera* is introduced, it can result in the competitive exclusion of *Apis cerana* (Ruttner 1988), depending on which subspecies are involved (e.g., Manila-Fajardo and Cleofas 2003).

We conservatively suggest that European Apini of the Miocene exhibited a considerable morphological diversity, even somewhat more so than in modern congeners. This is supported by the fact that among the specimens from Randeck, even those within morphotype D, showed a remarkable variation in body size, which cannot be explained solely by postmortem effects, by caste differences, or biological phenomena. The heterogeneity is further supported by the considerable variability in leg shape and the varying presence or absence of the small process of 1m-cu, all of which are apparently independent of the two morphotypes recognized on the basis of FWVA.

Noteworthy, our results show a much lower variability for the Oligocene Apini from Germany and France. As shown by the Honeybee Genome Sequencing Consortium (2006), the rate of evolution in *Apis* is slow compared to other insects. This may perhaps explain how European populations of *Apis* maintained such hyper-variability within an otherwise single evolutionary species for such a considerable time throughout the Miocene. Consequently, the various morphotypes observed across these European populations would perhaps all represent a single, widespread species, much like modern widespread species such as *Apis mellifera* and *Apis cerana*. The historical biogeography and nest evolution of the genus shall be discussed elsewhere (Kotthoff et al. in prep.).

## Appendix 1

Forewing venation angle measurements. (doi: 10.3897/zookeys.96.752.app1) File format: Microsoft Word.

Copyright notice: This dataset is made available under the Open Database License (http://opendatacommons.org/licenses/odbl/1.0/). The Open Database License (ODbL) is a license agreement intended to allow users to freely share, modify, and use this Dataset while maintaining this same freedom for others, provided that the original source and author(s) are credited.
